# On the Design of a Thermo-Magnetically Activated Piezoelectric Micro-Energy Generator: Working Principle

**DOI:** 10.3390/s22041610

**Published:** 2022-02-18

**Authors:** Adrian A. Rendon-Hernandez, Skandar Basrour

**Affiliations:** 1 Interdisciplinary Microsystems Group, University of Florida, Gainesville, FL 32611, USA; arendonhernandez@ufl.edu; 2 TIMA, University Grenoble Alpes, CNRS, Grenoble INP, 38000 Grenoble, France

**Keywords:** thermo-magnetization, piezoelectricity, time-varying temperature, miniaturization, energy harvesting

## Abstract

This paper deals with a new design of a thermo-magnetically activated piezoelectric generator. This proposed generator exploits the temperature-dependent magnetization of a ferromagnetic material, which is exposed to temporary change of temperature cycles. To promote a better understanding of the operation of this mechanism, a global coupled numerical model is presented, which is able to predict the static and dynamic behavior of the generator. It is shown that with some modifications to the physical design, the generator can be tuned for different activation temperatures. Energy densities of 280 and 67 µJcm^−3^ were achieved by the proposed model of the generator for its opening and closing commutation, respectively.

## 1. Introduction

Since the past decade there has been a growing interest in energy harvesters using piezoelectric materials [1,2,3]. Several studies, for instance [4,5,6], have been conducted on piezoelectric energy harvesters. These studies tended to focus on various orientations for the generator including inward, outward, and tilted configurations. Also, they demonstrated that design parameters like tilt angle and installation distance can be used to adjust the resonance frequency of the system to match the excitation frequency. More recent evidence [7,8] shows that flexible piezoelectric nanogenerators can be used to monitor body motions. By utilizing nonlinearity, force amplification mechanism and piezoelectric effect, energy harvesting integrated with wireless sensor is demonstrated. Although piezoelectric materials are widely used to directly convert mechanical energy into electrical energy, they can also be used in multi-step mechanisms for energy harvesting [9]. Thermal energy is present all around us either in the form of spatial thermal gradients or temporal changes of temperature. And every energetic process, regardless of its initial nature, namely, kinetic, electrical or chemical, often ends as heat that is then returned to environment. Several techniques for converting directly thermal energy into electricity have been studied, including thermoelectric generation and pyro-electricity [10,11,12,13,14]. Even if these techniques have the advantage of being direct conversion methods that suggests fewer conversion losses, they have some inconveniences when miniaturization is one of the design premises. Thermo-electric devices produce a relatively low voltage output in the presence of a small thermal gradient, and they need a heat sink facing a problematic to maintain an exploitable thermal gradient. Moreover, their power output and efficiency are relatively low for powering autonomous systems [15]. To overcome these inconveniences, some alternatives to direct thermal to electrical energy conversion have been proposed; that is double step thermal to mechanical to electrical energy conversion. The key principle behind conversion of thermal to mechanical energy is based on having a reversible and thermally dependent property like gas expansion (thermo-acoustics [16,17]), strain (shape memory alloy [12,13], thermal buckling [14,15]), or ferromagnetism (thermo-magnetic [16,17]). Thermal bimetallic devices have some disadvantages, for instances, they are not very accurate concerning the thermal behavior, they are difficult to calibrate, and they are not suitable for operating at lower temperature as the metals and metallic alloys show nearly same expansion or contraction in a lower range of temperatures [18]. A major challenge in designing multi-step energy conversion systems is the multiphysics complexity. Current solutions to predict multi-step energy conversions can suggest time-consuming simulation analysis and costly fabrication stages. Knowledge for modeling all design parameters to predict a device’s dynamic behavior is crucial to avoid expensive fabrication stages before design validation. In the following, we present a numerical model of the energy conversion processes in order to understand the influence of design parameters on power output. Thus, an innovative method of energy harvesting that exploits the temperature dependent magnetization of a ferromagnetic material is presented in this work. We take motivation from the work reported in [19] proposing an alternative to direct conversion of temperature fluctuations into electricity and the coupled multi-physics modeling of a thermal-gradient-to-electricity energy harvester presented in [20]. This paper seeks to promote better understanding on developing a complete multi-physics model for thermo-magnetically activated piezoelectric energy harvester. In addition, in this work we provide design rules aiming at miniaturizing the proof of concept presented in our previous work [21]. The novelty of this study refers to providing design rules for thermo-magnetically activated piezoelectric generators along with a coupled numerical model, which is intended to predict both the static and dynamic behavior of the generator through rapid simulations. One of the advantages of having a coupled model is the possibility to validate design parameters before starting any fabrication stage or time-costing finite-element analysis simulations. After having introduced the context and aims of this research, Section 2 will present the working principle of the proposed micro-energy generator. The analytical modelling and they mathematical equations are derived in Section 3. Section 4 concludes with the modeling validity and presents some suggestions for future work.

## 2. Materials and Methods

**Figure 1:**
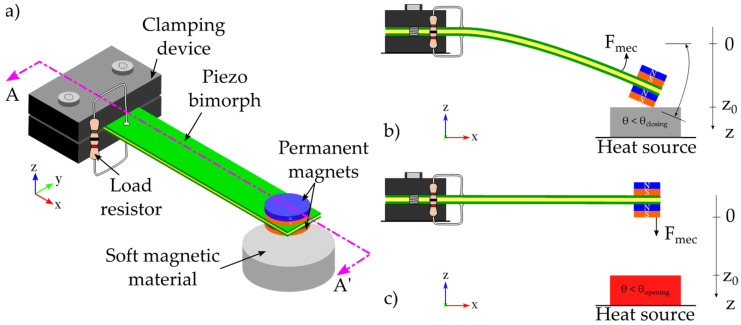
(**a**) Schematic view of the micro energy harvester showing its two stable operation positions: (**b**) closed position and (**c**) open position.

Figure 1 shows a schematic of a possible architecture of the proposed thermo-magnetically activated micro-generator. In this configuration, the generator can be divided into two main subsystems: the triggering system and the transduction one. The former is based on a thermo-magnetic circuit composed of a hard magnet and a soft magnetic alloy, for instance NdFeB and FeNi, respectively. Regarding the transducer system, it is composed of a cantilever beam made of a thin film of reinforcement material such as steel that is sandwiched between two thin films of piezoelectric layers, for instance lead zirconate titanate (PZT 5H). As regards of the piezoelectric beam, it consists of a bimorph PZT whose dimensions (L × W × H) are 20 × 4 × 0.3 mm. The soft magnetic material consists of an iron-nickel allow disc whose radius and thickness are 7.5 mm and 4 mm, respectively. A couple of permanent magnets whose radius and thickness are 2 mm and 1 mm, respectively, form the magnetic system. Even though we recognize that a design optimization needs to be performed in order to maximize the performance of the generator, we have estimated the size of the different elements by using the available soft magnetic material sample in our resources as the reference volume. Therefore, we have estimated the size for all other elements with respect to it; the piezoelectric beam was cut in order to achieve an acceptable deformation with respect to the size of both the permanent magnets and the soft magnetic material.

The hard magnets are attached at the free end of the cantilever beam whereas the soft magnetic alloy is centered and placed below the permanent magnets at a certain initial gap distance $z_{0}$ to be exposed to ambient temperature fluctuations. The operating mechanism of the harvester shown in Figure 1 is as follows: at the initial state, the soft magnetic alloy temperature is below its magnetic Curie temperature $\theta_{C}$; hence an attraction magnetic force $F_{mag}$ is exerted due to the permanent magnets. Thus, the permanent magnets are attracted towards the soft magnetic alloy and the free end of the beam travels towards the soft magnetic material surface making contact against each other under magnetic and inertial forces. At that instant, the generator is in the closed position (see Figure 1b). Once the ambient temperature increases; the soft magnetic material is now heated up by conduction and it loses its magnetism above its Curie temperature $\theta_{C}$. At the time that the temperature of the soft magnetic alloy approaches its Curie temperature, the attraction magnetic force drastically decreases below the mechanical reaction force *F_mec_* (i.e., $F_{mag}\;\leq {\;F}_{mec}$). After that, the counter-reaction force $F_{mec}$ of the cantilever beam pulls it away from the soft magnetic alloy. At that instant, the generator is in the open position (see Figure 1c). Next, when the ambient temperature decreases below the Curie temperature of the soft magnetic alloy, it takes back its spontaneous magnetization. After regaining magnetization, the permanent magnets are attracted towards the soft magnetic material and they travel towards the soft magnetic material surface making contact against each other. Temperature fluctuations on the soft magnetic alloy are driving the generator to either of its two stable operation positions. Since the attraction magnetic force is nonlinear, and dependent on both temperature and initial gap distance, it is possible to create a thermal threshold; a temperature value where the device shifts state. Actually, since the interaction between the hard-permanent magnets and the soft magnetic alloy drives the stable position of the generator, there are two thresholds, one for opening commutation $\theta_{opening}$ and the other for closing commutation $\theta_{closing}$. The operation commutations can continue as long as the ambient temperature fluctuations are maintained between the two thermal thresholds. Finally, the transduction system converts the kinetical mechanical energy on the cantilever beam during these two commutations.

One assumption is that the ambient temperature fluctuations are maintained such that the thermal thresholds (i.e., $\theta_{opening}$ and $\theta_{closing}$) are reached. Ambient temperature fluctuations can be caused by several reasons, like the waste thermal energy due to industrial machines or temperature fluctuations at the industrial pipes, which makes this model applicable to many scenarios. The mechanical stiffness of the cantilever beam is a very essential design parameter to determine the energy harvesting capabilities of the generator; it has an impact on strain energy due to the cantilever tip beam displacement and sets the restoring force required to overcome the magnetic attraction force to bring the cantilever beam back to its open position. The initial gap distance is also a crucial design parameter; it affects the attraction magnetic force exerted between the hard magnets and the soft magnetic alloy and it contributes to the initial elastic energy stored in the beam when the generator is at its closed position.

**Figure 2:**
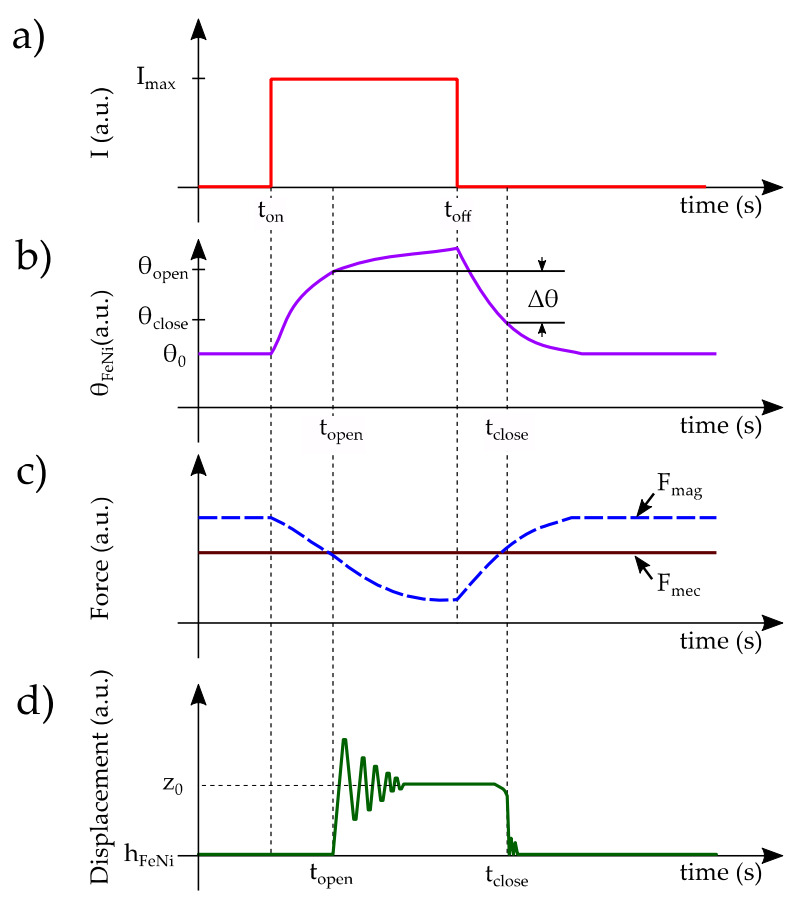
Schematic representation of the overall system operation during a thermal cycle; (**a**) direct current (DC) step driving the temperature changes on the soft magnetic alloy by means of Joule heat effect, (**b**) evolution of the temperature of the soft magnetic alloy showing both opening and closing thresholds, (**c**) magnetic and mechanical forces, *F_mag_* and *F_mec_*, respectively, are counterbalanced by temporal variations of temperature, and (**d**) vertical displacement of the cantilever tip within one cycle of operation.

The chronograms of Figure 2 show the typical response of the system when a thermal cycle is reached. It is evident to note that the hysteresis cycle created by this behavior corresponds to the minimum temperature span required for a complete operation of the generator.

**Figure 3:**
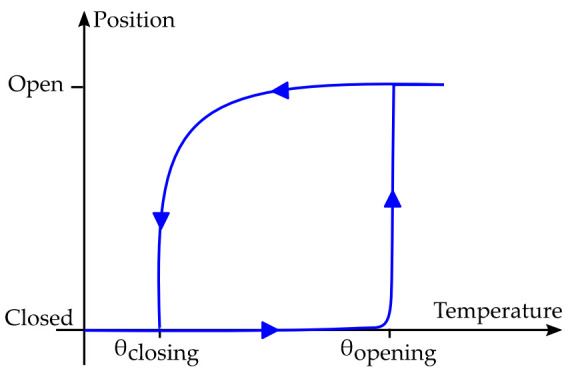
Representation of the natural evolution of the position of the generator as function of the thermal cycling.

It can be deduced that the opening commutation has an abrupt behavior, whereas the closing one is smooth. This is linked to the non-linearity of the magnetic attraction force, as shown in Figure 3.

## 3. Analytical Modeling

In this section, we present the analytical equations describing the operation of the generator. This operation can be divided into three distinct phases as follows: (i) Phase I—the permanent magnet at the bottom is in contact with the top surface of the soft magnetic alloy; thus, the generator is in its closed position. (ii) Phase II—a temperature increase on the soft magnetic alloy causes a loose of its spontaneous magnetization until reaching the opening threshold of the system, thus the opening commutation is reached resulting in an oscillatory motion of the beam. (iii) Phase III—the cantilever beam is now at its horizontal position and a temperature decreasing of the soft magnetic material makes it to regain its spontaneous magnetization up to reach the closing threshold of the system, therefore the closing commutation is achieved.

Phase I corresponds to the base configuration, where the initial deflection of the cantilever beam is required to provide a unidirectional restoring force in the direction opposing the unidirectional magnetic force. At this stage, the attraction magnetic force is higher than the mechanical restoring force. Phase II, the soft magnetic material heats up above its Curie temperature, losing its spontaneous magnetization; hence the attraction magnetic force decreases drastically, and it is no longer greater than the mechanical restoring force. Therefore, the cantilever beam is released, and it starts to oscillate with a significant amplitude and velocity. Phase III corresponds to the state where a decreasing of temperature in the soft magnetic material makes it to regaining its magnetization: the cantilever beam tip is pressed against the surface of the soft magnetic material under a stronger magnetic force than the mechanical force. In this phase, a rebound due to the contact between the hard magnet and the soft magnetic material surface is significant as well as the velocity. Below we provide mathematical description of each of these phases. 

### 3.1. Heat Transfer Model

In the design described herein, heat transfer occurs during heating up and cooling of the soft magnetic alloy. Initially, at the closed position of the generator, heating occurs at the heat source where the soft magnetic alloy and the cantilever beam tip are heated up by direct contact (i.e., conduction). Subsequently, at the open position of the generator, cooling of the system is achieved by either natural or forced convection through an oscillatory motion in air (i.e., convection). Equations for the time-dependent temperature at the upper face of the soft magnetic alloy, are formulated in order to describe heating and cooling processes.

We started with an unsteady energy balance on the soft magnetic alloy of mass, and specific heat. In Phase I, the system is in maximum bending displacement. In this state, thermal contact conductance dominates the heat transfer physics. In Phase II the role of thermal contact conductance is negligible and the temperature distribution changes only due to internal diffusion.

Since the purpose of heat transfer modelling is to compute the temperature in soft magnetic alloy and the corresponding magnetic force, the heat transfer can be divided into two parts: (i) heating up of generator at its closed position, which implies a solid-solid interface present only where two surfaces are in contact, and (ii) cooling of the generator at its open position through the interstitial fluid medium, which is always present or through a forced convection, like a fan for instance. Contact conductance causes conduction of heat through very small contact areas of the contacting surface that press against each other. The gap conductance is contributed by conduction through an interstitial fluid trapped between the contacting surface. It depends on the thermal conductivity, convection coefficient, of the interstitial fluid. In phases I and II, all thermal contact conductance mechanisms are present, while in phase III only the gap conductance due to the temperature. To model the heat transfer on the system, first we consider the generator of a determined shape of mass m, volume V, surface area $S_{L}$, density ρ, and specific heat $c_{p}$ as an isothermal body (i.e., interior temperatures remain essentially uniform during a heat transfer process), so we can treat it as a “lump” system whose temperature is only a function of time, θ(t). Initially, the generator is at equilibrium with its environment, suddenly exposed to a heat source, that depending on the heat flow direction, it can be a conductive heat source: when increasing of temperature takes place (soft magnetic material heating up), or a convective heat source: when decrease of temperature occurs (soft magnetic material cooling up). To produce temperature variations on our soft magnetic material specimen, we assumed it is always in contact with a heat source. This heat source consists in electrical energy being converted into heat at a rate of ${R\cdot i}^{2}$ (*R* and *i* correspond to the electrical resistance and current for the Joule heating effect, respectively). This electrical current is flowing across a serpentine-shaped printed circuit board, which is attached to the bottom surface of the soft magnetic alloy. From a steady-state energy balance, the time rate of change of the energy content of a given mass is equal to the rate at which energy is added to the mass plus rate of production of energy within the mass. An energy balance of the generator for a time interval d*t* can be expressed as follows:

(1) $$mc_{p}\frac{d\theta }{dt}=\left[Ri^{2}-\lambda_{conv}S_{L}\left(\theta -\theta_{amb}\right)\right]$$

After a classical solution of the ordinary differential equation and considering the initial condition $\theta \left(0\right)=\theta_{amb}$ (ambient temperature) we find the governing equation of the system during the heating up stage:

(2) $$\theta \left(t\right)=\theta_{amb}+\frac{Ri^{2}}{\lambda_{conv}S_{L}}\left(1-e^{-\frac{\lambda_{conv}S_{L}}{mc_{p}}t}\right)$$

where $\theta_{amb}$ is the ambient temperature, m the mass of the soft magnetic material including the transducer beam as it is in contact with the metal alloy, and $c_{p}$ is the specific heat of the system.

During the heating process, the surface area is composed of the whole system as the contact between the magnet and the soft magnetic alloy is achieved. The heat transfer stops as soon as the electrical current leaves the circuit.

For cooling up stage, the energy balance can be derived as follows:

(3) $$\frac{d\theta }{dt}=-\frac{\omega_{fan}\dot{m}\left(\theta -\theta_{amb}\right)}{60m}-\frac{\lambda_{conv}S_{L-FeNi}\left(\theta -\theta_{\infty }\right)}{mc_{p}}$$

This equation describes the cooling process using forced convection of the soft magnetic alloy that is not in contact against the hard magnet (i.e., the surface area is only that one of the soft magnetic alloy). The first term of the right-hand side represents the forced convection. The terms $\omega_{fan}$ and $\dot{m}$ are the fan speed (revolutions per minute) and mass flow rate (kilogram per second), respectively. When natural convection is considered, this first term is zero. The second term represents the convection interaction of the system. At this point, the surface area $S_{L-FeNi}$, is only that one of soft magnetic alloy because the transducer beam is no longer in contact with it. After a classical solution of this ordinary differential equation considering the initial condition $\theta \left(0\right)=\theta_{i}$ the governing expression for the cooling stage with forced convection can be written as follows:

(4) $$\theta =\theta_{\infty }+e^{-\frac{\left(\omega_{fan}c_{p}+60\lambda_{conv}S_{L-FeNi}\right)t}{60mc_{p}}}\left(\theta_{i}-\theta_{\infty }\right)$$

An important outcome from Equations (2) and (4) is the thermal time constant of the two heat transfer stages; $\tau_{heating}$ (heating up stage) and $\tau_{cooling}$ (cooling down stage):

(5) $$\tau_{heating}=\frac{mc_{p}}{\left(\lambda_{conv}S_{L}\;\right)}$$

If natural convection is considered; the following equation describes the time constant for the cooling phase of the generator:

(6) $$\tau_{cooling}=\frac{mc_{p}}{\left(\lambda_{conv}S_{L-FeNi}\;\right)}$$

As the surface area is lower when the generator is open compared with the closed generator, it suggests that a size reduction of the device can lead lower constant of time for both heating up and cooling stages. Basically, these terms are strongly linked to the dimensions of the generator and they impact directly on the time needed to pass from one commutation to another. Therefore, the lower are the dimensions of the generator the lower are the time period between commutations. When considering forced convection, this constant of time is dramatically reduced because of the influence of the heat source considered (i.e., the fan). In order to explore this model, we considered some initial parameters that are summarized in Table 1.

**Figure 4:**
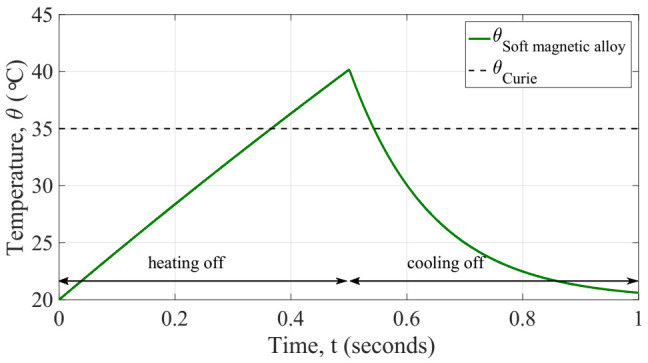
Simulated temperature response of the generator (soft magnetic alloy) during both heating up and cooling stages.

Considering the aforementioned parameters, we evaluated the response in temperature of the model and results are depicted in Figure 4.

### 3.2. Thermo-Magnetic Model

The triggering system is composed of an iron nickel alloy, which is a soft magnetic material with a Curie temperature near to room temperature, it depends on material stoichiometry. A neodymium iron boron permanent magnet is a commercially magnet available in several shapes and sizes. It has a remanent flux density ($B_{r}\;≅1.3\;T$ at room temperature) and a relatively high Curie temperature (310 °C), and it can retain its magnetization within the working range of ambient temperatures. The neodymium iron boron magnet is of a cylindrical configuration, and its magnetic flux density can be expressed according to Equation (7) [18]

(7) $$B_{mag}\left(z\right)=\frac{B_{r}}{2}\left[\frac{z+\left(2h_{mag}\right)}{\sqrt{{\left(z+2h_{mag}\right)}^{2}+r_{mag}^{2}}}-\frac{z}{\sqrt{z^{2}+r_{mag}^{2}}}\right]\;$$

Here, $B_{r}$, $h_{mag}$ and $r_{mag}$ are the remanent flux density, thickness and radius of the neodymium iron boron magnet, respectively. It is magnetized along the thickness direction, which corresponds to the z-axis coordinate system, z is the distance away from the magnet surface, and $B_{mag}\left(z\right)$ is the corresponding magnetic induction at location z. This induction subjects the soft magnetic alloy (FeNi) to an external magnetic field intensity denoted by $H_{mag}\left(z\right)=B_{mag}\left(z\right)/\mu_{0}$, where $\mu_{0}$ is the magnetic permeability of vacuum. Depending on the temperature within the material, soft magnetic alloy follows a corresponding magnetization distribution. Estimating the magnetization of such a soft magnetic material requires solving the equation given as [19]:

(8) $$M_{s}\left(\theta \right)=M_{s}{}_{\left(0\right)}{\left(1-\frac{\theta }{\theta_{c}}\right)}^{\beta }$$

**Figure 5:**
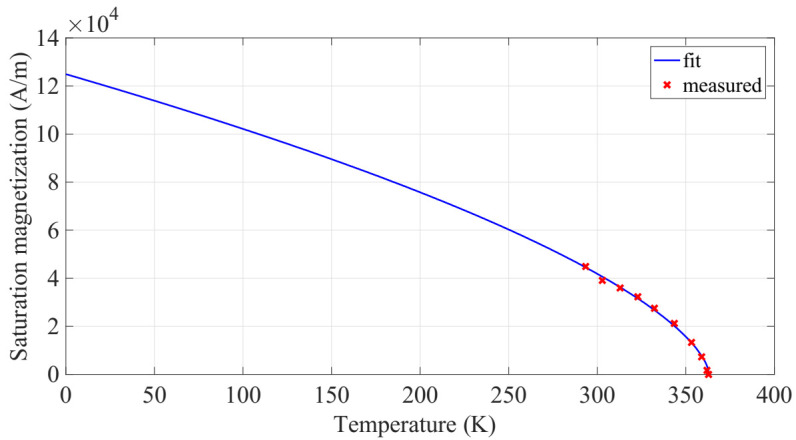
Measured saturation magnetization against temperature in FeNi material and its curve fitting from [20].

Here, $M_{s\left(0\right)}$ is the value of saturation magnetization at 0 K, $\theta_{c}$ is the Curie temperature, and β is the material-dependent critical exponent, a representative of mean field interactions. Mavrudieva et al. [20] measured the saturation induction $B_{s}$ as function of temperature on a FeNi sample, that was elaborated by the Research Center of IMPHY ALLOYS (Imphy, France). Considering that $M=B_{r}\mu_{0}^{-1}$, the measured saturation induction was used to identify $M_{s}\left(0\;K\right)$ and β by curve fitting as shown in Figure 5.

The change from the ferromagnetic to the paramagnetic state is perfectly sharp at the Curie temperature, $\theta_{c}=363.15\;K$. The calculated values of parameters through fitting technique are summarized in Table 2.

Since the configuration of the triggering system is such as having an attraction magnetic force between the permanent magnet and the soft magnetic material, we have interest in calculating this actuation force.

### 3.3. Magnetic Force

On the design here, the commutations are driven by the interaction of the restoring mechanical force (i.e., created by the cantilever piezoelectric beam) and the magnetic attraction force acting on the soft magnetic material (i.e., caused by the applied magnetic field $B_{ext}=B_{mag}\left(z\right)$ due to the neodymium magnet). Therefore, it is important to understand what design parameters affect these forces and how to derive them. With regards the magnetic force, it may be apparent from Equation (7) that the remanent flux density $B_{r}$ of the neodymium magnet, its radius and thickness are $r_{mag}$ and $h_{mag}$, respectively, and the distance z away from it determines the external magnetic field at that location. Also, the magnetic attraction force is dependent on the volumetric and surface magnetic charge densities on the soft magnetic material, both of which are functions of the local magnetization and its dimensions. Magnetization of the soft magnetic material is a function of temperature, as shown in Figure 5. Hence, after solving the heat transfer model and getting the temperature of the generator, the magnetic force can be calculated.

If a magnetized volume $V_{FeNi}$ is subjected to an applied field $H_{mag}$, it acquires a magneto-static potential energy [18]:

(9) $$E_{mag}=\int_{V}^{}MB_{mag}dV$$

where M is the fixed magnetization due to the continuum of dipole moments of which the specimen is composed of. The magneto-static force can be calculated as the gradient of the potential energy. In other terms, this magnetic force can be expressed by a volume integral of the magnetic material and the gradient of magnetic flux density $B_{mag}$ and magnetization M as shown in Equation (10):

(10) $$\vec{F_{mag}}=\vec{\nabla }\left(E_{mag}\right)=\int_{V}^{}\vec{\nabla }\left(MB_{mag}\right)dV$$

This can be simplified for the case that the magnetic volume (i.e., the soft magnetic alloy) is perpendicular to the symmetry axis of the permanent magnet:

(11) $$F_{mag}\left(z\right)=V_{FeNi}\left(M\frac{\partial B_{mag}}{\partial x}+M\frac{\partial B_{mag}}{\partial y}+M\frac{\partial B_{mag}}{\partial z}\right)\approx V_{FeNi}M_{s}\frac{\partial B_{mag}}{\partial z}$$

**Figure 6:**
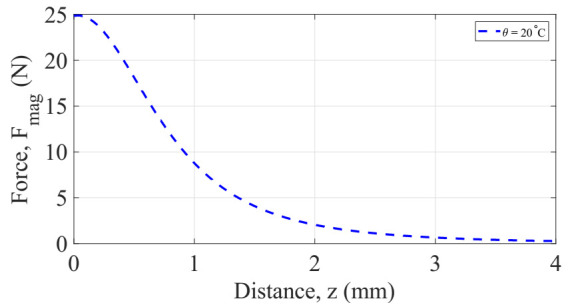
Absolute value of the attraction magnetic force on the triggering system as function of the separation distance z at room temperature (293 K), thickness and radius of magnet are, $h_{mag}=1\;\mathrm{mm}$ and $r_{mag}=1\;\mathrm{mm}$, respectively.

In addition, assuming an alignment between the permanent magnet and the soft magnetic alloy, we can approximate the magnetic attraction force as a function of the gap distance and temperature using Equations (8) and (11):

(12) $$F_{mag}\left(z,\theta \right)\approx V_{FeNi}\left[M_{s\left(0\right)}{\left(1-\frac{\theta }{\theta_{c}}\right)}^{\beta }\left\{\frac{B_{r}}{2}r_{mag}^{2}\frac{{\left[{\left(z+2h_{mag}\right)}^{2}+r_{mag}^{2}\right]}^{\frac{3}{2}}-{\left(r_{mag}^{2}+z^{2}\right)}^{\frac{3}{2}}}{{\left[{\left(z+2h_{mag}\right)}^{2}+r_{mag}^{2}\right]}^{\frac{3}{2}}{\left(r_{mag}^{2}+z^{2}\right)}^{\frac{3}{2}}}\right\}\right]$$

where, $B_{r}$, $h_{mag}$, and $r_{mag}$, are the remanent flux density (Tesla), thickness (m), and radius (m), respectively of the neodymium iron boron magnet. $V_{FeNi}$ and $M_{s}$ are the volume and saturation magnetization, respectively, of the soft magnetic material. The latter is dependent on temperature. $F_{mag}$ is the corresponding attraction magnetic force over the soft magnetic material at location z. This approximation of the magnetic force is illustrated in Figure 6 for a model considering the parameters listed in Table 3. It is shown the non-linearity of the magnetic force as function of the distance z.

**Figure 7:**
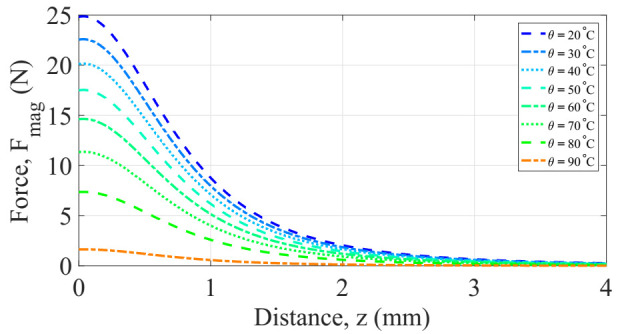
Evolution of the magnetic force as function of temperature and distance separation between magnet and soft magnetic alloy.

From Equation (8), the saturation magnetization $M_{s}$ of the soft magnetic alloy is dependent on the temperature, as shown in Figure 5. Thus, it may be apparent from Equation (12) that the magnetic force is a function of: the distance z separation between the magnets and the soft magnetic alloy, the magnetization and the temperature θ. $F_{mag}\left(z\right)$ was computed numerically using Matlab. The results are shown in Figure 7.

The parameters considered for obtaining these results are summarized in Table 4. Initially we thought that the attraction magnetic force was null when the temperature is above the point of Curie of the soft magnetic material. However, a more careful analysis revealed that even at the temperature of Curie, the soft magnetic specimen can develop a certain amount of magnetic force when is subject to the external flux density due to the permanent magnets. As shown in Figure 7, at the temperature of Curie of this soft magnetic volume, there exists a magnetic force but is no significant compared with that one of the restoring mechanical force. Thus, the generator can be driven to its two operation positions with the balance of these two forces. Moreover, from these results, it can be concluded that increasing the temperature of the soft magnetic alloy results in decreasing the magnetic force; similarly, for the dependence on the gap distance.

At this point, we have already derived the equations for one of the two actuation forces, calculation of the counter-reaction force is described below.

### 3.4. Vibration Model

The vibration of the piezoelectric cantilever beam under the attraction magnetic force can be described as a second order mass-spring-damper system, along with a piezoelectric element connected parallel to the damper. The governing equation with the addition of suitable damping, can be written, as follows:

(13) $$m\frac{d^{2}z}{dt^{2}}+C\frac{dz}{dt}+kz=F_{mag}$$

where m, C, and k are the effective mass, total damping coefficient, and effective stiffness of the system, respectively. Mechanical force is depending on the electro-mechanical coupling, which can be described as follows:

(14) $$F_{mec}=k\left(z_{0}-z\right)+\alpha V_{piezo}\;i=\alpha \frac{dz}{dt}-C_{0}\frac{dV_{piezo}}{dt}$$

where k is the mechanical equivalent stiffness of the cantilever beam, α is the force factor of the piezoelectric material in $Nm^{-1}$, $V_{piezo}$ is the piezoelectric generated voltage, and $C_{0}$ is the equivalent capacitance of the piezoelectric elements. The coupled system between the mechanical vibration and the electrical domain is as follows:

(15) $$F_{mag}=m\frac{d^{2}z}{dt^{2}}+C\frac{dz}{dt}+k\left(z_{0}-z\right)-\alpha V_{piezo}\;i=\alpha \frac{dz}{dt}-C_{0}\frac{dV_{piezo}}{dt}$$

This equation system is iteratively solved using Matlab (ver. R2017a, Natick, MA, USA). More details are presented on the next section.

### 3.5. Global Coupling Model

Before discussing the different effects governing the vibration, let us first understand the physics by just considering one complete thermal cycle. Firstly, given an initial design for the generator, its operation temperatures $\theta_{opening}$ and $\theta_{closing}$ have to be derived. The method we used for computing these parameters is in line with a variation of the same as that used by Korvink *et al.* [21] for an electrostatic parallel-plates actuator and its pull-in voltage and travel. With some adjustments. First, we estimated the total balance force in the system. Since the actuation forces on the generator are $F_{mag}$ and $F_{mec}$, we are interested in finding the stable position for this interaction. Thus, the resultant force can be described as follows:

(16) $$F_{T}=F_{mec}-F_{mag}=0$$

Using Equations (12) and (14), we can rewrite the force balance as follows:

(17) $$k\left(z_{0}-z\right)=\frac{1}{2}V_{FeNi}M_{s}B_{r}\left\{r_{mag}^{2}\frac{{\left[{\left(z+2h_{mag}\right)}^{2}+r_{mag}^{2}\right]}^{\frac{3}{2}}-{\left(r_{mag}^{2}+z^{2}\right)}^{\frac{3}{2}}}{{\left[{\left(z+2h_{mag}\right)}^{2}+r_{mag}^{2}\right]}^{\frac{3}{2}}{\left(r_{mag}^{2}+z^{2}\right)}^{\frac{3}{2}}}\right\}$$

After arranging the previous equation, let’s call the auxiliary function h(z):

(18) $$\frac{V_{FeNi}M_{s}B_{r}}{2k}=\frac{\left(z_{0}-z\right){\left[{\left(z+2h_{mag}\right)}^{2}+r_{mag}^{2}\right]}^{\frac{3}{2}}{\left(r_{mag}^{2}+z^{2}\right)}^{\frac{3}{2}}}{r_{mag}^{2}\left\{{\left[{\left(z+2h_{mag}\right)}^{2}+r_{mag}^{2}\right]}^{\frac{3}{2}}-{\left(r_{mag}^{2}+z^{2}\right)}^{\frac{3}{2}}\right\}}\;h\left(z\right)=\frac{\left(z_{0}-z\right){\left[{\left(z+2h_{mag}\right)}^{2}+r_{mag}^{2}\right]}^{\frac{3}{2}}{\left(r_{mag}^{2}+z^{2}\right)}^{\frac{3}{2}}}{r_{mag}^{2}\left\{{\left[{\left(z+2h_{mag}\right)}^{2}+r_{mag}^{2}\right]}^{\frac{3}{2}}-{\left(r_{mag}^{2}+z^{2}\right)}^{\frac{3}{2}}\right\}}$$

**Figure 8:**
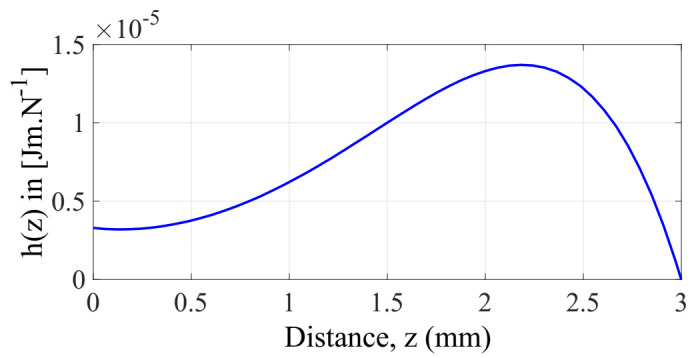
Representation of the auxiliar function $h\left(z\right)$ for a given an initial gap $z_{0}=3\mathrm{mm}$, $r_{mag}=1\;\mathrm{mm}$ and $h_{mag}=1\;\mathrm{mm}$.

The plot of this auxiliar function h(z), for a given value of $z_{0}$ and $h_{mag}$ (magnet thickness) is shown in Figure 8.

Now, to find the maximum value of the function h(z) we apply the criterion of the first derivative ∂h(z)/∂z=0. The resulting expression was then treated to find its roots within the interval $0\leq z\leq z_{0}$. To do that, we fixed the parameters $h_{mag}=1\;\mathrm{mm}\;$ and $r_{mag}=1\;\mathrm{mm}$ to evaluate the function for $1.5\;\mathrm{mm}\leq z_{0}\leq 5\;\mathrm{mm}$ with a step size of 0.5 mm. From Figure 8 and after some numerical approximations it was found the maximum point of the function h(z), which tends to $\left(z_{0}/\;\surd 2\right)$. Now, we can evaluate Equation (18) at this point (i.e., $h\left(z_{0}/\surd 2\right)$). The resulting expression is now inserted on its right-hand side giving the following expression:

(19) $$\frac{V_{FeNi}M_{s}B_{r}}{2k}=h\left(\frac{z_{0}}{\surd 2}\right)\;\frac{V_{FeNi}M_{s}B_{r}}{2k}=\frac{z_{0}\left(\sqrt{2}-2\right)ABC}{8r_{mag}^{2}\left(A-BC\right)}$$

Here, A, B, and C are defined by Equation (20):

(20) $$A=\left(2r_{mag}^{2}+z_{0}^{2}\right){\left(4r_{mag}^{2}+2z_{0}^{2}\right)}^{\frac{1}{2}}\;B=\left(4z_{0}\sqrt{2}h_{mag}+8h_{mag}^{2}+2r_{mag}^{2}+z_{0}^{2}\right)\;C={\left(2z_{0}^{2}+8z_{0}\sqrt{2}h_{mag}+16h_{mag}^{2}+4r_{mag}^{2}\right)}^{\frac{1}{2}}$$

After some simplifications and solving Equation (19) for $M_{s}$, we obtain an expression as function of the initial design parameters, which give the saturation magnetization for the opening commutation of the generator. Finally, we use this equation into Equation (8) to find the temperature of opening as follows:

(21) $$\theta_{opening}=\theta_{C}\langle 1-e^{\frac{\ln\left\{-\frac{\left(\sqrt{2}-2\right)kz_{0}ABC}{4r_{mag}^{2}B_{r}V_{FeNi}M_{s\left(0\right)}\left[\left(4\sqrt{2}h_{mag}z_{0}+8h_{mag}^{2}+2r_{mag}^{2}+z_{0}^{2}\right)C-\left(2r_{mag}^{2}+z_{0}^{2}\right)D\right]}\right\}}{\beta }}\rangle \;A=\left(2r_{mag}^{2}+z_{0}^{2}\right){\left(4r_{mag}^{2}+2z_{0}^{2}\right)}^{\frac{1}{2}}\;B=\left(4z_{0}\sqrt{2}h_{mag}+8h_{mag}^{2}+2r_{mag}^{2}+z_{0}^{2}\right)\;C={\left(2z_{0}^{2}+8z_{0}\sqrt{2}h_{mag}+16h_{mag}^{2}+4r_{mag}^{2}\right)}^{\frac{1}{2}}\;D={\left(4r_{mag}^{2}+2z_{0}^{2}\right)}^{\frac{1}{2}}$$

Similarly, making the balance of actuation forces when the generator is at its open position. In other words, it is now required an attraction magnetic force greater than the maximal mechanical force to ensure the closing commutation. Thus, the temperature for closing commutation is given by:

(22) $$\theta_{closing}=\theta_{C}\langle 1-e^{\frac{\ln\left\{\frac{2kz_{0}EF}{r_{mag}^{2}B_{r}V_{FeNi}M_{s\left(0\right)}\left(F-E\right)}\right\}}{\beta }}\rangle \;E={\left(r_{mag}^{2}+z_{0}^{2}\right)}^{\frac{3}{2}}\;F={\left(4h_{mag}^{2}+4z_{0}h_{mag}+r_{mag}^{2}+z_{0}^{2}\right)}^{\frac{3}{2}}$$

**Figure 9:**
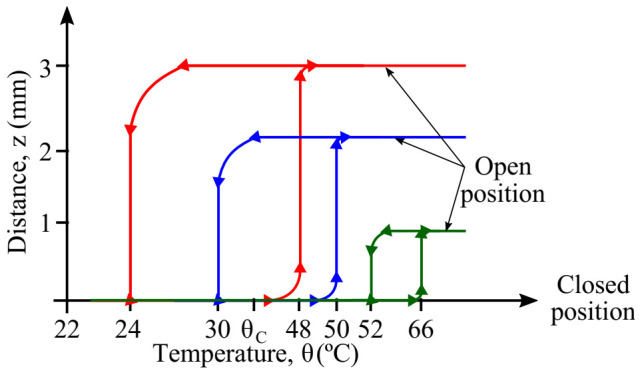
Experimental results showing the impact of the initial gap distance $z_{0}$ on the commutation temperatures for a given design of the generator. The FeNi sample has a Curie temperature around 35 ± 5 °C.

At this point, we are able to derive the thermal thresholds given for an initial design of the generator. To better understand the quasi-static behavior of the generator, we conducted experimental measurements on a prototype for which we fixed the parameters like the dimensions of soft magnetic material, transducer bimorph and magnets. The only parameter we changed was the gap distance z between the magnet and the soft magnetic material. We opted for three different values of this parameter $z_{0}$: 1, 2, and 3 mm, as shown in Figure 9. For each initial gap distance, the experiment was repeated under conditions in which the ambient temperature was about 22 °C and the maximum temperature at which the soft magnetic material was heated was 80 °C. Through a triangulation technique using a laser sensor, we monitored the vertical displacement at the free end of the cantilever beam all the time during the experiment. This first set of experiments confirmed the impact of the initial gap distance on both the operation temperatures and the thermal hysteresis. The latter describes the separation between the commutation temperatures for a given design of the generator.

These tests revealed that it is possible to tune the operation temperatures by adjusting the gap initial distance, moreover, to obtain commutation temperatures near to environment, higher initial gaps must be considered. Even though the fact that this leads to greater thermal hysteresis, reducing the volume of the soft magnetic material can dramatically also reduce the transient temperature response to pass from one commutation temperature to another. Making an energy conversion balance of the system, it may be apparent that the initial deflection of the beam is represents the maximum possible output energy that can be converted into electricity, thus greater initial gaps are desired. Added to this; lower thermal hysteresis is expected in order to reduce the transition period between commutations. This is why we believe that we found analytical model of such a system able to predict its quasi-static and dynamic behaviors in function of their design parameters. Let us first apply the coupled model in order to obtain in static behavior the operating temperatures of such a design. Considering the initial design of generator listed in Table 5.

## 4. Results and Discussion

**Figure 10:**
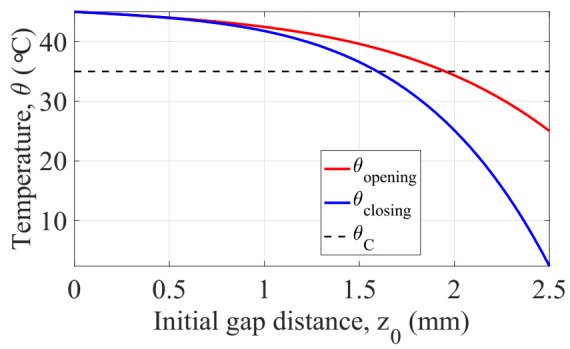
Operating temperatures representation as function of initial gap distance for a giving equivalent stiffness $k=350\;Nm^{-1}$.

According with the initial design of the generator listed in Table 5, the equivalent stiffness of the cantilever beam according to the model detailed in [22] was calculated. The operation temperatures were obtained through Equations (21) and (22) and are shown in Figure 10. We decided to make a parametric study on initial gap distance from 0 to 2.5 mm in order to explore the design space available for different input scenarios.

The static behavior of the generator comprises the operation for which the system passes from the closed position to the open one and vice versa. This is critical for designing and giving a first estimation of the performance that can be achieved by the device under a specific scenario. By reducing the amount of soft magnetic material, the magnetic force is also reduced, however, it implies lower constants of time for the temperature transient response of the device. In addition, to compensate the weakness of this magnetic force due to reduction of the soft magnetic material volume, a lower equivalent stiffness of the generator has to be considered. This can be reached by several ways: (i) modifying the thickness of the piezoelectric layers, (ii) adjusting the length of the piezoelectric layers, (iii) reducing the volume of the magnets, by reducing the mass of the magnet, it reduces the effective stiffness of the transducer, which in turn implies a greater oscillation frequency of the transducer, thus higher amount of kinetic energy that can be converted into electricity during the open commutation.

### Generator Dynamic Behavior

**Figure 11:**
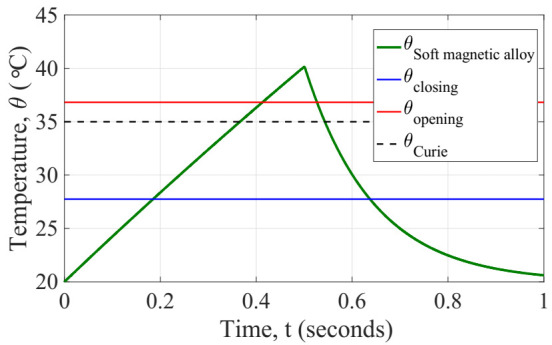
Temperature response of the generator showing the operating temperatures with reference to the Curie temperature of the soft magnetic alloy.

**Figure 12:**
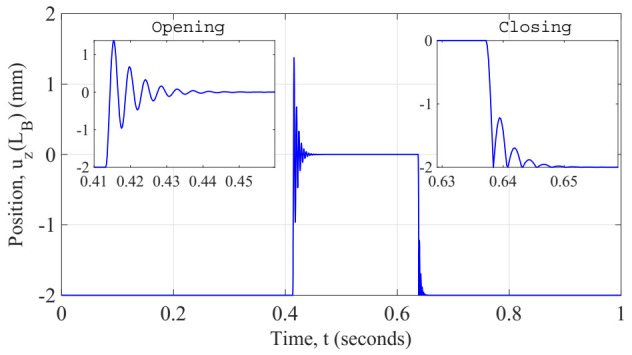
Simulated response of the displacement at the tip of the beam.

In order to investigate the dynamic performance of the initial design of generator, we conducted a transient simulation of the coupled system solving iteratively the equations aforementioned. We opted for implementing this mathematical model in Matlab (ver. R2017a, Natick, MA, USA) for solving the coupled system iteratively with a time step of 200 µs to check the mechanical vibration of the beam during its commutations. First, the temperature response of the system was explored, as shown in Figure 11. At the initial time of simulation, the electrical current is applied and maintained constant up to the half of the time simulation end. The whole system is at ambient temperature 20 °C, thus at its closed position. Once the simulation time reaches the half period, the current is turned off and simultaneously the fan is turned on to get a forced convection over the generator. This state continues until the end of the simulation. We did monitor some important output variables of the generator over the simulation time, for instance: the temperature of the soft magnetic alloy, it confirms the quasi-static prediction of the generator, the operating temperatures of the system (which are expected to remain constant as the design is also constant), the tip cantilever beam displacement, as shown in Figure 12. These values support the kinetic performance of the generator, and the output voltage on the terminals of the load resistor as well as the instantaneous power dissipated by the load resistor. These results are described below.

As expected, the initial position of the generator was at the closed position; thus, the contact between the soft magnetic material and the permanent magnet is done at the initial gap distance (i.e., −2 mm with respect to the horizontal plane of the cantilever beam without deflection). This simulation result revealed that the frequency of the oscillations of the cantilever beam when the opening commutation is 220 Hz. However, the value analytically estimated in a static behavior was 225 Hz. It is because of the damping linked to the magnetic attraction force that is still present on the system even when the cantilever beam is released (at the opening commutation the magnetic attraction force is not enough to maintain the contact but to reduce the oscillations of the cantilever beam). We believe that this difference emphasizes the validity of our model. To describe the effect of the rebound when the closing commutation occurs, we adopted the approach of reinitialize the velocity of the tip cantilever beam to a new value at the instant the tip beam reaches the contact (i.e., the saturation limit, initial gap distance $z_{0}$). In our model, this method implies that when the magnet hits the soft magnetic alloy; its velocity can be set to a different value, i.e., to the velocity after the impact. Moreover, to capture the velocity of the tip cantilever beam just before the collision, a memory block is then used to calculate the velocity after the impact. The coefficient of restitution e is introduced as the ratio of the final to initial relative velocity between two objects after they collide. It normally ranges from 0 to 1 where 1 would be a perfectly elastic collision. For nickel alloys, this coefficient oscillates between 0.15 and 0.7, we assume a value of 0.5.

The resulting vibration behavior of the cantilever beam can be validated as its velocity is zero for the phases where the generator is closed position and after it reaches steady state; it is after oscillations when releasing and impact due to contact. In addition to the strain on the cantilever beam, these velocity responses would suggest that during the opening commutation another kind of transduction, electromagnetic for instance, could be implemented to extract electrical energy. It is desired to get greater deformations on the cantilever beam as this conduces to greater elastic energy initially stored on the generator. Furthermore, the influence of the equivalent stiffness on the natural frequency of the beam dictates that is suitable having higher frequencies as this increases the amount of energy during the opening commutation. This is why, a balance of these design parameters is then listed in Table 6.

As can be seen from the above table, the reducing the volumes of the generator device leads to better response in heat transfer. This reduction of the volume has to be done considering the rest of design rules listed in order to respect the operation restriction of the generator.

**Figure 13:**
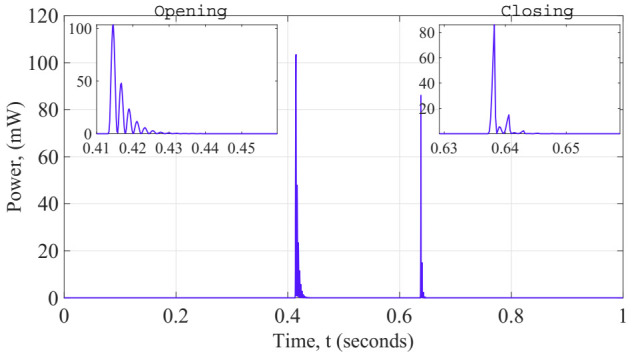
Instantaneous power response during transient simulation.

Solving for the electrical energy harvesting requires solving a coupled system with an additional electrical boundary condition. The piezoelectric bimorph is considered to working linearly for the working range of the model; any internal resistance on the piezoelectric layers were ignored because of the lower value compared with the load resistance connected. The effect of the presence of the magnetic field on the electrical charge was neglected. A load resistor of 8 kΩ was connected to the electrodes of the cantilever beam. This finding provides additional support for understanding the dynamic behavior of the generator. The harvested power corresponds to the dissipated power on the load resistance. The instantaneous power dissipate electrical power is then calculated as shown in Figure 13.

Since the input scenario where we planned to place this generator is uncertain and random, we decided to explore the performance of our generator in terms of energy density per commutation. In other words, for a worst case, when the temperature fluctuations allow the generator at least one thermal cycle of commutation (i.e., opening commutation and closing commutation). The effective volume considered is that generated when the cantilever beam reaches the maximum displacement towards the closed state and the opening one. Whereas for the average power per commutation it was estimated taking into account the duration time of 10 cycles of the fundamental oscillation of the cantilever beam, as shown in Equation (23):

(23) $$E=\int_{0}^{10T}p_{ave}dt$$

Here, T represents the period of the natural oscillation of the cantilever beam, $p_{ave}$ is the average power dissipated by the load resistor within a duration of 10T per commutation of the generator. This approach is significant due to the possibility of comparing, based on the same criterion, different design generator’s performance. The estimated energy densities per commutation of this initial design are listed in Table 7:

## 5. Conclusions

In this paper, a coupled-multiphysics analysis of a thermo-magnetically activated micro-generator has been presented. The result of a particular scenario has been demonstrated. This method represents a viable alternative to rapid design of such a micro-generator. Analyses including a heat transfer model, thermo-magnetic model, magnetic force, vibration model and global coupled model, were developed and used to examine the influence of various design parameters on the generator power output. The temperature response of the generator was first investigated. Time constants for the generator heating up and cooling down stages were calculated. After, the thermomagnetic response was studied revealing the generator operation temperatures as a function of the gap between the magnet and soft magnetic material. By using the proposed numerical model, the dynamic behavior of then generator was predicted and a set of design guidelines proposed. Moreover, numerical simulation results suggested energy densities up to 280 µJ·cm^−3^ and 67 µJ·cm^−3^, for opening and closing commutation, respectively. The multi-physics numerical model we presented here is capable of predicting a range of different scenarios of temperature fluctuations for which; the generator is supposed to work in. It also provides an opportunity to examine the interactions of several physic effects acting on each of the generator subsystems. Furthermore, it also provides an opportunity to study the influence of the device parameters on the dynamic performance of the generator. Increasing the initial gap distance may lead to greater elastic energy initially stored on the cantilever beam. Thus, resulting in greater energy harvesting capabilities. However, for greater initial gap distances, the counter-reaction force is increasing as well, thus, the required attraction magnetic force to ensure the closed position is greater as well, which can easily exceed the magnetic properties of the triggering system. This finding suggests an optimal design for which the energy balance could be maximized. Concerning the operation temperatures of the generator, they are depending on the design parameters, essentially on the triggering system including the initial gap position and the resulting effective stiffness of the cantilever beam. Hence, it is possible to tuning these thermal thresholds by modifying design parameters as the dimensions or the initial gap distance, which can be an advantage if the material selection is restricted. The larger the gap between magnets and soft magnetic material, the larger the beam deflection and consequently, it indicates larger range between operating temperature. Conversely, in order to reduce the range between operating temperatures, a reduction in the gap has to be set up. The thermo-magnetically activated piezoelectric micro-generator reported here shows a promising approach. It can operate through ambient temperature fluctuations or industrial environmental temperature variations depending on the tuning of their thermal thresholds. It could harvest small and slow temperature fluctuations present around us: walls of pipes carrying hot fluids, industrial machines, automobile radiators; to name but a few. Moreover, it can play a second role, as a thermal switch.

## Figures and Tables

**Table 1:** General parameters for the temperature response simulation of the generator.

Parameter	Symbol	Units	Value
Surface area of device	$S_{L}$	$m^{2}$	$234\times 10^{-6}$
Surface area of soft magnetic alloy	$S_{L-FeNi}$	$m^{2}$	$154\times 10^{-6}$
Mass	m	kg	$3.7\times 10^{-3}$
Specific heat	$c_{p}$	$Jkg^{-1}K^{-1}$	505
Electrical current	i	A	2
Electrical resistance	R	Ω	20
Heat transfer coefficient	$\lambda_{conv}$	$Wm^{-2}K^{-1}$	28
Fan speed	$\omega_{fan}$	rpm	2000
Mass flow rate	$\dot{m}$	$kgs^{-1}$	$0.5\times 10^{-3}$

**Table 2:** Identification results for saturation magnetization against temperature.

Parameter	Symbol	Units	Value
Saturation magnetization at 0 K	$M_{s\left(0\right)}$	$Am^{-1}$	$1.249\times 10^{5}$
Critical exponent	β	-	0.625
Curie temperature	$\theta_{c}$	K	363.15

**Table 3:** General parameters of the permanent magnets for the temperature response.

Parameter	Symbol	Units	Value
Remanent flux density of magnet	$B_{r}$	T	1.15
Thickness of magnet	$t_{mag}$	m	$1\times 10^{-3}$
Radius of magnet	$r_{mag}$	m	$1\times 10^{-3}$
Permeability of vacuum	$\mu_{0}$	$Hm^{-1}$	$4\pi \times 10^{-7}$

**Table 4:** General parameters of the soft magnetic material for calculating the magneto-static force as function of temperature and gap distance.

Parameter	Symbol	Units	Value
Thickness of soft magnetic material	$h_{FeNi}$	mm	4
Radius of soft magnetic material	$r_{FeNi}$	mm	7.5
Saturation magnetization at 0 K	$M_{s\left(0\right)}$	$Am^{-1}$	$4.1\times 10^{4}$

**Table 5:** General parameters of the initial design of the generator for calculating the operation temperatures in quasi-static behavior.

System	Parameter	Symbol	Units	Value
Transducer system cantilever beam	Length of cantilever beam	$L_{B}$	mm	20
	Width of cantilever beam	$w_{B}\;$	mm	4
	Thickness of shim layer	$h_{p}$	µm	100
	Young’s modulus of shim layer	$Y_{p}$	MPa	100
	Poisson’s ratio of shim layer	$\nu_{B}$	-	0.3
	Density of shim layer	$\rho_{B}$	$kgm^{-3}$	7500
Transducer system piezoelectric material	Length of piezoelectric layer	$L_{p}$	mm	20
	Thickness of piezoelectric layer	$h_{p}$	µm	105
	Stiffness of electrical field	$S_{xx}^{E}$	$10^{-12}Pa^{-1}$	20.5
		$S_{xz}^{E}$	$10^{-12}Pa^{-1}$	−8.45
		$S_{xy}^{E}$	$10^{-12}Pa^{-1}$	−4.78
		$S_{zz}^{E}$	$10^{-12}Pa^{-1}$	13.45
	Piezoelectric coefficient	$d_{xz}$	$pCN^{-1}$	−274
	Permittivity at constant strain	$\varepsilon_{zz}^{S}$	$nFm^{-1}$	30.1
	Permittivity at constant stress	$\varepsilon_{zz}^{T}$	$nFm^{-1}$	27.7
Triggering system soft magnetic alloy	Radius of soft magnetic alloy	$r_{FeNi}$	mm	7.5
	Thickness of soft magnetic material	$h_{FeNi}$	mm	4
	Density of soft magnetic alloy	$\rho_{FeNi}$	$kgm^{-3}$	6000
	Curie temperature	$\theta_{C}$	°C	35
	Critical exponent	β	-	0.625
	Specific heat of soft magnetic material	$c_{FeNi}$	$Jkg^{-1}K^{-1}$	505
	Saturation magnetization at 0 K	$M_{S\left(0\right)}$	$Am^{-1}$	$1.25\times 10^{5}$
	Heat transfer coefficient	$\lambda_{conv}$	$Wm^{-2}K^{-1}$	28
	Fan speed	$\omega_{fan}$	rpm	2000
	Mass flow rate	$\dot{m}$	$gs^{-1}$	0.5
	Initial gap distance	$z_{0}$	mm	2
Triggering system permanent magnet	Radius of magnet	$r_{mag}$	mm	2
	Thickness of magnet	$h_{mag}$	mm	1
	Density of magnet	$\rho_{mag}$	$kgm^{-3}$	5000

**Table 6:** Design guidelines of a thermo-magnetically activated piezoelectric generator.

Criterion	Operation	Design Parameter of Impact	Design Rule
Volume of soft magnetic material	Provides thermal dependent magnetization	$F_{mag}$	It has to be strong enough to counter $F_{mec}$
		$\tau_{heating}$	They have to be as low as possible to reduce the time between commutations
		$\tau_{cooling}$	
Volume of magnets	Provides external flux density to induce $F_{mag}$	$h_{mag}$ $r_{mag}$	An optimal ratio has to be selected to ensure a functional $F_{mag}$
	Acts as seismic mass	k	It shall be as large as possible to increase natural oscillation
Volume of piezoelectric transducer	Piezoelectric layers dimensions	$L_{P}$ $,\;h_{P}$ $,\;x_{P}$	An optimal dimension should be considered to maximize the output voltage. As they affect directly the stiffness of the system, thus $F_{mec}$
	Shim layer dimensions	$L_{B}$ $,\;w_{B}$ $,\;h_{B}$	Thickness of layers have greater impact on the system stiffness than the other parameters.
	Changes the surface area of the generator when it is closed	$S_{L}$	Reducing the lateral surface of the generator can reduce $\tau_{heating}$ and $\tau_{cooling}$, thus reduce as well as time between commutations.
Initial gap distance	Adjusts the operating temperatures $\theta_{opening}$ and $\theta_{closing}$	$z_{0}$	It has to be as large as possible to increase the stroke of displacement on the cantilever beam, thus higher elastic energy to be converted into electricity
	Scales the mechanical restoring force of the transducer beam	$F_{mec}$	By increasing $z_{0}$$,\;F_{mec}$ is then increased, which obliges to adjust the magnetic attraction force to get the operation commutation in a feasible range of temperature

**Table 7:** Energy densities per commutation of the initial design of generator.

Energy Density per Commutation	Symbol	Units	Value
Opening commutation	$ED_{opening}$	$\mu Jcm^{-3}$	280
Closing commutation	$ED_{closing}$	$\mu Jcm^{-3}$	67

## Data Availability

Not applicable.
